# A phase Ib study of pertuzumab, a recombinant humanised antibody to HER2, and docetaxel in patients with advanced solid tumours

**DOI:** 10.1038/sj.bjc.6604043

**Published:** 2007-11-13

**Authors:** G Attard, J Kitzen, S P Blagden, P C Fong, L C Pronk, J Zhi, G Zugmaier, J Verweij, J S de Bono, M de Jonge

**Affiliations:** 1 Drug Development Unit, The Royal Marsden NHS Foundation Trust, Sutton, UK; 2 Erasmus MC, Department of Medical Oncology, Rotterdam, The Netherlands; 3 F. Hoffmann-La Roche Ltd., Division Pharma Oncology, Basel, Switzerland

**Keywords:** pertuzumab, docetaxel, phase I, HER dimerisation inhibitors

## Abstract

Pertuzumab represents the first in a new class of targeted therapeutics known as HER dimerisation inhibitors. We conducted a phase Ib study to determine the maximum-tolerated dose, the dose limiting toxicities (DLT), and pharmacokinetic (PK) interaction of docetaxel when administered in combination with pertuzumab. Initially, two dose levels of docetaxel (60 and 75 mg m^−2^) were explored in combination with a fixed dose of 1050 mg of pertuzumab; then two dose levels of docetaxel (75 and 100 mg m^−2^) were explored in combination following a fixed dose of 420 mg of pertuzumab with a loading dose of 840 mg. Both drugs were administered intravenously every 3 weeks. The latter dose of pertuzumab was allowed after an amendment to the original protocol when phase II data suggesting no difference in toxicity or activity between the 2 doses became available. Two patients out of two treated at docetaxel 75 mg m^−2^ in combination with pertuzumab 1050 mg suffered DLT (grade 3 diarrhoea and grade 4 febrile neutropaenia). Two out of five patients treated at docetaxel 100 mg m^−2^ in combination with pertuzumab 420 mg with a loading dose of 840 mg suffered DLT (grade 3 fatigue and grade 4 febrile neutropaenia). Stable disease was observed at four cycles in more than half of the patients treated and a confirmed radiological partial response with a >50% decline in PSA in a patient with hormone refractory prostate cancer were observed. There were no pharmacokinetic drug–drug interactions. The recommended phase II dose of this combination was docetaxel 75 mg m^−2^ and 420 mg pertuzumab following a loading dose of 840 mg.

The ErbB or human epidermal growth factor receptor (HER) family of receptor tyrosine kinases are important mediators of cell growth, survival, and differentiation (Sundaresan *et al*, 1998). Although 11 ligands are known to bind to various HER family members, none of these ligands binds directly to the human epidermal growth factor receptor 2 (HER2). Instead, HER2 functions as a coreceptor and ligand-driven heterodimerisation of HER2 with other HER family members appears to play an important role in neoplastic transformation and/or progression. Studies in cell culture systems show that heregulin-activated HER3-HER2 heterodimers elicit the strongest proliferative and transformation responses of any possible receptor combination (Pinkas-Kramarski *et al*, 1996). Pertuzumab (Omnitarg™) is a fully humanised monoclonal antibody, which acts by blocking the association of HER2 with other HER family members, including the epidermal growth factor receptor, HER3 and HER4 (Adams *et al*, 2006). As a result, pertuzumab inhibits ligand-initiated intracellular signalling through two major signal pathways: mitogen-activated protein kinase and phosphatidylinositol 3 kinase. Inhibition of these signalling pathways can result in cell growth arrest and apoptosis, respectively (Hanahan and Weinberg, 2000).

Pertuzumab is being developed for the treatment of patients with solid tumours (Gordon *et al*, 2006; Johnson and Janne, 2006; Walshe *et al*, 2006). Although both pertuzumab and trastuzumab target HER2, they bind to distinct epitopes, and consequently, ligand-activated downstream signalling induced by heterodimerisation is blocked by pertuzumab but not by trastuzumab. As a result of these differences, pertuzumab may inhibit the growth of tumours that do not overexpress HER2. In phase I studies, pertuzumab was well tolerated with the most common adverse events being fatigue, nausea, and vomiting (Agus *et al*, 2005). Although, non-acneiform skin rash and diarrhoea were observed, these were mainly grade 1, only occasionally grade 2 and did not appear to be dose related (Laux *et al*, 2006). The maximum tolerated dose was not reached with dose escalation to 15 mg kg^−1^. Pharmacokinetic studies indicated a terminal elimination half-life of 2–4 weeks supporting 3-weekly fixed dosing (Ng *et al*, 2006). Docetaxel is an antimicrotubule agent that enhances polymerisation of tubulin into stable microtubules and inhibits microtubule depolymerisation. This leads to a disruption of the equilibrium within the microtubule system and ultimately leads to cell death (Rowinsky and Donehower, 1991). Docetaxel is an active drug in various solid tumours and is also an attractive agent for incorporation into combination regimens. *In vivo* studies on the combination of pertuzumab with various cytotoxic agents, including docetaxel, have demonstrated that there is at least an additive anti-tumour effect without compromising the toxicity profile. The potential improvement in anti-tumour activity to be gained by combining pertuzumab with docetaxel, and the minimal overlap in toxicity profiles, led to the conduct of this phase 1b study designed to determine the maximum-tolerated dose of pertuzumab and docetaxel when administered in combination every 21 days. The secondary objectives were to assess the safety and tolerability of this combination, to evaluate if there was any pharmacokinetic interaction between pertuzumab and docetaxel and to determine the objective responses in advanced solid tumours.

## PATIENTS AND METHODS

### Patients

Patients with histologically confirmed advanced solid tumours that had progressed during or after standard therapy or for which no standard therapy was available were eligible for this study. A minimum of 4 weeks had to have passed from prior treatment with chemotherapy or radiotherapy. Patients were also required to have a life expectancy of at least 12 weeks and a Eastern Co-operative Oncology Group (ECOG) performance status of 0 or 1. Other eligibility criteria included adequate bone marrow (absolute neutrophil count ⩾1500 m^−3^, platelet count ⩾100 000 m^−3^, and haemoglobin (Hgb) ⩾9 g dl^−1^), renal (creatinine ⩽upper normal limit or creatinine clearance of ⩾60 ml min^−1^), hepatic (bilirubin ⩽upper normal limit and aspartate aminotransferase [AST], and alanine aminotransferase [ALT] ⩽2.5 times the upper limits of normal) and cardiac (baseline left ventricular ejection fraction [LVEF] of ⩾50%) function and a serum calcium within normal limits. Similarly, patients were excluded from the study if they had uncontrolled hypertension, symptomatic CNS metastasis or neuropathy ⩾grade 2 according to NCI-CTC version 3.0, or any prior malignancy, cardiac condition or serious medical illness that would affect their management according to the study protocol. Patients were also excluded if they had received a prior cumulative doxorubicin dose greater than 360 mg m^−2^ or equivalent or had a prior history of severe hypersensitivity reactions to polysorbate 80. The institutional review boards at both participating sites approved the study protocol, and written informed consent was obtained before any study-related procedures.

### Study design and treatment

This was a phase Ib, open-label, two-center study. Pertuzumab and docetaxel were administered as an intravenous (IV) infusion every 3 weeks. Pertuzumab was provided by F Hoffmann-La Roche (Basel, Switzerland). Each 10 ml single-use vial contained 175 mg of pertuzumab formulated in 10 mmol l^−1^ L-histidine (pH6.0), 240 mmol l^−1^ sucrose, and 0.02% polysorbate 20. The first dose of pertuzumab was given by intravenous infusion over 90 min. If the initial infusion was well tolerated, the infusion time was reduced to 30 min for subsequent infusions. Initially, the planned dose of pertuzumab was fixed dose at 1050 mg for each dose level. However, based on results of phase II single-agent studies in metastatic breast cancer (unpublished data), ovarian cancer (Gordon *et al*, 2006), and hormone refractory prostate cancer (HRPC) (De Bono *et al*, 2007) which became available midway through this study and suggested no difference in toxicity or efficacy between the 420 and 1050 mg dose levels of pertuzumab, the protocol was amended to fix the dose of pertuzumab at 420 mg with a loading dose of 840 mg in cycle 1. The dose of docetaxel was sequentially escalated from 60 to 75 to 100 mg m^−2^ as outlined in Table 1. The doses of pertuzumab and docetaxel were reduced, and/or treatment cycles delayed, in patients experiencing dose-limiting toxicities. Initially, three patients were treated in cohort 1, with the first patient being monitored for at least 2 weeks before additional patients were treated. Escalation to a new dose level was permitted when at least two of three patients had been evaluated for 3 weeks. If dose limiting toxicities (DLT) were observed during cycle 1 in one of three patients, an additional three patients were to be treated at that dose level. At least six patients were to be treated at the 75 and 100 mg m^−2^ dose levels before a recommended phase II dose was declared. If patients withdrew for reasons other than toxicity before completing only one cycle, they were to be replaced at the same dose level. DLT was defined as one of the following adverse events: (1) non-hematological toxicity ⩾grade 3 including diarrhoea (except alopecia, and/or nausea and vomiting in patients who had not received optimal treatment with antiemetics); (2) grade 4 neutropaenia lasting at least 7 days or neutropaenia complicated by fever or infection regardless of duration, thrombocytopenia <25 × 10^9^ l^−1^ or any thrombocytopenia requiring platelet transfusion, and (3) any subjectively intolerable toxicity felt to be related to either one of the compounds. If DLT was observed in ⩾2 patients of six patients at a dose level, the maximum-tolerated dose had been exceeded and at least six patients would be accrued at the lower dose level to confirm that this was the maximum-tolerated dose.

### Analysis of pharmacokinetics

Pharmacokinetic (PK) assesments were performed in all patients to determine if there was any PK interaction between pertuzumab and docetaxel. As no dose-toxicity relationship had been demonstrated for pertuzumab in the phase I study (Agus *et al*, 2005), it was assumed that the administration of docetaxel would not lead to a clinically relevant modification of the exposure to pertuzumab. Therefore, PK assessments were focused on the potential modification of docetaxel exposure by pertuzumab. In the first treatment cycle, blood withdrawal for PK analysis was performed 15 and 30 min prior to administration of docetaxel and 15 and 30 min and 1, 2, 4, 8 and 23 h, post administration. Pertuzumab was administered at least 24 h after, on day 2, followed by PK analysis at 15 min and 1.5, 4 and 8 h. In the second cycle, pertuzumab was administered on day 1, immediately followed by the administration of docetaxel and sampling for PK analysis was repeated for both drugs. Plasma samples of docetaxel were analysed using a validated specific liquid chromatography-mass spectrometry (LC-MS/MS) method as described previously (Agus *et al*, 2005). Serum samples were assayed for pertuzumab concentrations using a receptor-binding, enzyme-linked immunosorbent assay. The assay uses immobilised antigen p185^HER2^ ECD to capture pertuzumab from serum samples. Bound pertuzumab was detected with mouse anti-human Fc-horseradish peroxidase (Jackson ImmunoResearch Laboratories Inc., West Grove, PA, USA), and tetramethyl benzidine (KPL Inc., Gaithersburg, MD, USA) used as the substrate for colour development to quantify serum pertuzumab against a known standard curve. The assay has a minimum quantifiable concentration of 0.25 *μ*g ml^−1^ for pertuzumab in human serum. Pharmacokinetic parameters for docetaxel and pertuzumab were estimated using non-compartmental methods (WinNonLin software version 5.0.1).

### Tumour assessments

The response evaluation criteria in solid tumours (RECIST) were used to assess objective response, time to disease progression, and duration of response (Therasse *et al*, 2000). Tumour burden was evaluated at baseline by physical examination and imaging including computed tomography of chest, abdomen and pelvis, and bone scans when clinically indicated. Responses were assessed by identical techniques at the end (week 3) of cycles 2 and 4, and every two cycles subsequently. Objective responses were confirmed by repeat assessments after ⩾4 weeks. Prostate-specific antigen (PSA) declines or rises were described in terms of the PSA working group criteria (Bubley *et al*, 1999) and CA-125 was measured in all ovarian cancer patients and described as a percentage change from baseline as described previously (Rustin *et al*, 2004).

### Tolerability and safety

The incidence and severity of all adverse events, changes in vital signs, laboratory assessments, physical examination findings, and medical conditions during and after treatment with pertuzumab were assessed at least weekly during the first two cycles, and at least weekly three times thereafter, and graded according to NCI-CTC Version 3. Laboratory evaluations included full blood count with differential (weekly two times) and serum electrolytes, creatinine, AST, ALT, and bilirubin before the start of every cycle. Continued close monitoring of cardiac function was performed, using noninvasive cardiac monitoring (two-dimensional echocardiography and Doppler or Multi-Gated Acquisition scans) every 6 weeks and reported as a change from baseline. In addition, electrocardiographs were obtained at screening and follow-up, and serum markers of cardiac damage (troponin T) were collected every cycle.

## RESULTS

### Patient characteristics

A total of 19 patients (13 male), with a median age of 59 years (range 22–69 years), were enrolled between April 2004 and July 2005. Nine patients (47%) had HRPC and three patients (10%) had ovarian cancer reflecting the anticipated anti-tumour activity of pertuzumab and docetaxel in these two tumour types. All patients with prostate cancer were refractory to LHRH analogues and anti-androgens and five (56%) had also developed progressive disease while taking steroids. Seven of the patients with HRPC were chemotherapy naive; 10 patients treated on this study had received a median of 2 lines (range, 1–6) of prior cytotoxic chemotherapy. Patient demographics, baseline characteristics, and prior anti-cancer treatments are listed in Table 2.

### Safety

A total of 99 treatment cycles were administered to 19 patients, with a median of 5 cycles per patient (range, 1–15 cycles). No DLTs were observed in the first three patients treated in cohort 1. The first two patients included in cohort 2 treated at 75 mg m^−2^ of docetaxel and 1050 mg of pertuzumab developed DLTs, comprising of grade 4 febrile neutropaenia (neutrophil count 0.4 × 10^9^ l^−1^) on day 8 of the first cycle in one patient with grade 3 diarrhoea on day 4 of the first cycle in another, lasting for 2 days resolved without sequelae after treatment with loperamide, and grade 3 fatigue on day 6 in a second patient. As per protocol an additional three patients were treated in cohort 1. None of these patients developed a DLT. After a protocol amendment six patients were recruited to cohort 2A. No DLTs were observed and five patients were subsequently recruited and treated in cohort 3A. Two of these patients developed DLTs in cycle 1 (grade 4 febrile neutropaenia and grade 3 fatigue) and recruitment was discontinued. A third patient treated in cohort 3A had grade 3 stomatitis in cycle 2 requiring dose reduction. The grade 3 and 4 toxicities observed are summarised in Table 3. No infusion-related reactions were observed. Of the 17 patients with baseline and post-baseline cardiac function evaluations, three patients (18%) experienced an asymptomatic LVEF decrease from baseline of ⩾10%: two patients in cohort 2A, one by 14% from 62 to 48% and the other from 61 to 48%, and one patient in cohort 1, from a baseline of 77% to a value of 53%. The former patient experienced recovery of LVEF to 53% on repeat investigation but the latter two patients had no improvement at final visit.

### Pharmacokinetics

**Figure 1:**
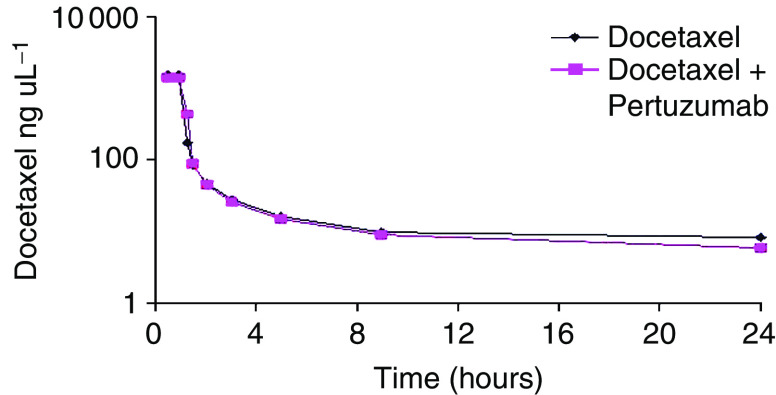
Plasma concentration time curves for docetaxel 75 mg m^−2^ monotherapy and combined with pertuzumab.

Pharmacokinetic parameters for pertuzumab (in the presence of docetaxel), for 1050 mg in cycles 1 and 2 and for 840 mg cycle 1 and 420 mg cycle 2 were comparable with single agent values of pertuzumab from other studies at the same doses (Table 4). The AUC_0–last_ was greater for cycle 2 in keeping with accumulation of drug. The *C*_max_, AUC and clearance values for docetaxel alone and in combination with pertuzumab were very similar (Table 5) and correlated with overlapping plasma concentration time curves as demonstrated in Figure 1, indicating no drug–drug interaction.

### Anti-tumour activity

Fourteen (82.4%) of 19 patients had stable disease by RECIST criteria after two cycles of treatment and nine (47.3%) had stable disease after four cycles. One patient with HRPC treated in cohort 1 had stable disease by both PSAWG criteria and RECIST for 13 cycles at which time his PSA increased by >25% from baseline although his CT scan showed stable disease. One patient with HRPC treated in cohort 2A had a partial response by RECIST criteria after 4 cycles, confirmed after 8 cycles of treatment. He also had a decline in PSA by >50%. One patient in each of cohorts 2, 2A, and 3A had a confirmed PSA decline >50% in the presence of stable disease by RECIST criteria. One ovarian cancer patient treated in cohort 1 had a 47% decrease in CA-125 levels from baseline after four cycles of treatment at which time she had stable disease by RECIST and elected to stop treatment.

## DISCUSSION

This study reports that the combination of pertuzumab and docetaxel is safe with manageable side-effects. Grade 4 febrile neutropaenia, grade 3 diarrhoea, and grade 3 fatigue were reported DLTs. Grade 3 stomatitis in cycle 2, necessitating a dose reduction, was observed in one patient. PK results from this study have shown that when docetaxel and pertuzumab were concomitantly administered, there was no apparent change in their PK. The PK parameters were consistent with those observed in data published previously (Bruno *et al*, 1998) and therefore, no drug–drug interaction is expected. The toxicity profile of the combination was similar to what would be expected with docetaxel alone and a dose for the combination for future studies has been identified.

This study was not designed to identify whether an improvement in the anti-tumour activity of docetaxel can be achieved by combining this with pertuzumab. Nonetheless, stable disease at four cycles in more than half of the patients treated, supports further phase II evaluation of this combination in specific tumour types. Significant anti-tumour activity has been reported with single-agent pertuzumab in refractory ovarian cancer (Gordon *et al*, 2006) and due to renewed interest in the anti-tumour activity of docetaxel in this tumour type (Blagden and Kaye, 2005), further investigation of docetaxel in combination with pertuzumab in ovarian cancer patients is warranted. Ovarian cancer was however under-represented in this study compared to HRPC in which no evidence of anti-tumour activity was reported in a single-agent phase II study (De Bono *et al*, 2007). Pertuzumab is also being investigated in metastatic breast cancer in combination with trastuzumab (Walshe *et al*, 2006) and in non small cell lung cancer (Johnson and Janne, 2006), both tumour types in which docetaxel is effective. Furthermore combination of pertuzumab and docetaxel with agents targeting epidermal growth factor receptor and other HER family members could overcome resistance secondary to functional redundancy in the ErbB network (Reid *et al*, 2007) and further studies of this combination with anti-tumour activity as a primary endpoint are anticipated.

## Figures and Tables

**Table 1:** Dose escalation and dose limiting toxicity

**Dose level**	**Pertuzumab (mg)**	**Docetaxel (mg m^−2^)**	**Number of patients planned (treated)**	**Number of patients with DLT**
*Initial dose escalation schedule*				
1	1050	60	3–6 (6)	0
2	1050	75	6 (2)	2
3	1050	100	6 (0)	—
				
*Amended dose escalation schedule:*				
1A	840/420	60	—	—
2A	840/420	75	6 (6)	0
3A	840/420	100	6 (5)	2

**Table 2:** Patient characteristics

**Characteristics**	**Total 19 patients**
*Age (years)*	
Median	59
Range	22–69
	
*Sex male/female*	13/6
	
*ECOG PS*	
0	9
1	10
	
*Tumour type*	
Prostate	9
Ovarian	3
Melanoma	2
Head and neck	1
Pancreas	1
Breast	1
Retroperitoneal paraganglioma	1
Lung	1
	
*Prior anticancer treatment*	
Cytotoxic chemotherapy	10 (53%)
○1 line	4 (21.1%)
○2 lines	2 (10.5%)
○3 lines	2 (10.5%)
○4 lines	1 (5.3%)
○6 lines	1
Hormonal therapies	13 (68%)

**Table 3:** Summary of grade 3 and 4 toxicities

	**Dose level**				
	**Cohort 1 (*N*=6)**	**Cohort 2 (*N*=2)**	**Cohort 2A (*N*=6)**	**Cohort 3A (*N*=5)**	**All cohorts (*N*=19)**
*All body systems*					
Number of patients with at least one grade 3 or 4 toxicity	3	2	2	4	11 (58%)
					
*Gastrointestinal disorders*					
Diarrhoea		2^a^	2^a^		4
Nausea				1	1
Abdominal Pain				1	1
Vomiting				1	1
Stomatitis				1^a^	1
					
*General disorders*					
Fatigue	1^a^	1	1^a^	1^a^	4
					
*Nervous system disorders*					
Neuropathy	1^b^				1
					
*Infections*					
Lower respiratory tract infection	1				1
Febrile neutropaenia		1^a^		1^a^	2
Candidiasis		1			1
Cystitis				1	1
Urinary tract infection	1				1
					
*Psychiatric disorders*					
Depression	1				1

a Adverse event considered to be related to the study medication.

b Paralysis secondary to brain metastasis.

**Table 4:** Summary of PK parameters for pertuzumab in combination with docetaxel

**Dose (mg)**	**Cycle**	***t*_1/2_ (day) Mean (range)**	***C*_max_ (*μ*g ml^−1^) mean (range)**	**AUC_0-last_ (*μ*g day ml^−1^) mean (range)**	**AUC_0-∞_ (*μ*g day ml^−1^) Mean (range)**	**Vss (ml) mean (range)**	**Cl (ml per day) mean (range)**
1050 *n*=8	1	13.36 (7.80–19.59)	301 (143–394)	2390 (1733–3355)	3951 (2348–4980)	5214 (2975–7036)	282 (211–447)
1050 *n*=7	2	22.08 (9.02–49.63)	368 (253–463)	3500 (3010–4346)	6856 (4521–11426)	4672 (3023–6580)	167 (92 232)
840 *n*=11	1	12.13 (6.95–25.96)	255 (169–447)	1749 (1214–3083)	2796 (1604–5116)	5355 (3028–8217	329 (164–524)
420 *n*=10	2	19.10 (8.85–42.00)	150 (102–233)	1491 (914–2289)	2762 (1639–3827)	4233 (2823–7058)	169 (110–256)

**Table 5:** Summary of PK parameters for docetaxel alone and in combination with pertuzumab

**Cohort**	**Dose group**	**Cycle**	***t*_1/2_ (h) mean (range)**	***C*_max_ (ng ml^−1^) mean (range)**	**AUC_0-∞_ (ng h ml^−1^) mean (range)**	**Vss (× 10^4^ ml m^−2^) mean (range)**	**Cl (× 10^3^ ml h m^−2^) mean (range)**
1	Docetaxel 60 mg m^−2^ alone *n*=6	1	16.9 (7.31–22.7)	1642 (1250–1960)	1838 (1523–2175)	79 (42–100)	33 (28–39)
1	Docetaxel 60 mg m^−2^+Pertuzumab 1050 mg *n*=6	2	19.7 (12.5–27.1)	1695 (1320–2270)	1734 (1512–2562)	102 (64–154)	36 (23–40)
2A	Docetaxel 75 mg m^−2^ alone *n*=6	1	12.7 (9.56–19.4)	3128 (2210–4580)	3744 (2386–5783)	41 (18–70)	22 (13–31)
2A	Docetaxel 75 mg m^−2^+Pertuzumab 420 mg *n*=6	2	15.2 (9.62–23.8)	2722 (1480–3830)	3496 (2178–5113)	57 (20–110)	24 (15–34)
3A	Docetaxel 100 mg m^−2^ alone *n*=5	1	9.59 (7.49–13.5)	5450 (3160–7740)	5930 (3522–8364)	25 (16–32)	18 (12–28)
3A	Docetaxel 100 mg m^−2^+Pertuzumab 420 mg *n*=4	2	12.8 (8.04–21.4)	4705 (2210–6570)	5218 (2409–7823)	43 (16–76)	22 (12–41)

## References

[bib1] Adams CW, Allison DE, Flagella K, Presta L, Clarke J, Dybdal N, McKeever K, Sliwkowski MX (2006) Humanization of a recombinant monoclonal antibody to produce a therapeutic HER dimerization inhibitor, pertuzumab. Cancer Immunol Immunother 6: 717–727 10.1007/s00262-005-0058-x PMC11030689 16151804

[bib2] Agus DB, Gordon MS, Taylor C, Natale RB, Karlan B, Mendelson DS, Press MF, Allison DE, Sliwkowski X, Lieberman G, Kelsey SM, Fyfe G (2005) Phase I clinical study of pertuzumab, a novel HER dimerization inhibitor, in patients with advanced cancer. J Clin Oncol 23: 2534–2543 15699478 10.1200/JCO.2005.03.184

[bib3] Blagden SP, Kaye SB (2005) Docetaxel in the management of ovarian cancer. Expert Rev Anticancer Ther 5: 203–214 15877518 10.1586/14737140.5.2.203

[bib4] Bruno R, Hille D, Riva A, Vivier N, ten Bokkel Huinnink WW, van Oosterom AT, Kaye SB, Verweij J, Fossella FV, Valero V, Rigas JR, Seidman AD, Chevallier B, Fumoleau P, Burris HA, Ravdin PM, Sheiner LB (1998) Population pharmacokinetics/pharmacodynamics of docetaxel in phase II studies in patients with cancer. J Clin Oncol 16: 187–196 9440742 10.1200/JCO.1998.16.1.187

[bib5] Bubley GJ, Carducci M, Dahut W, Dawson N, Daliani D, Eisenberger M, Figg WD, Freidlin B, Halabi S, Hudes G, Hussain M, Kaplan R, Myers C, Oh W, Petrylak DP, Reed E, Roth B, Sartor O, Scher H, Simons J, Sinibaldi V, Small EJ, Smith MR, Trump DL, Wilding G (1999) Eligibility and response guidelines for phase II clinical trials in androgen-independent prostate cancer: Recommendations from the Prostate-Specific Antigen Working Group. J Clin Oncol 17: 3461–3467 10550143 10.1200/JCO.1999.17.11.3461

[bib6] De Bono JS, Bellmunt J, Attard G, Droz JP, Miller K, Flechon A, Steinberg C, Parker C, Zugmaier G, Gimenez V, Cockey L, Mason M, Graham J (2007) An open label phase II study to evaluate the efficacy and safety of two doses of pertuzumab in castrate chemotherapy-naïve patients with hormone refractory prostate cancer. J Clin Oncol 25: 257–262 17235043 10.1200/JCO.2006.07.0888

[bib7] Gordon MS, Matei D, Aghajanian C, Matulonis UA, Brewer M, Fleming GF, Hainsworth JD, Garcia AA, Pegram MD, Schilder RJ, Cohn DE, Roman L, Derynck MK, Ng K, Lyons B, Allison DE, Eberhard DA, Pham TQ, Dere RC, Karlan BY (2006) Clinical activity of pertuzumab (rhuMAb 2C4), a HER dimerization inhibitor, in advanced ovarian cancer: potential predictive relationship with tumor HER2 activation status. J Clin Oncol 24: 4324–4332 16896006 10.1200/JCO.2005.05.4221

[bib8] Hanahan D, Weinberg RA (2000) The hallmarks of cancer. Cell 100: 57–70 10647931 10.1016/s0092-8674(00)81683-9

[bib9] Johnson BE, Janne PA (2006) Rationale for a phase II trial of pertuzumab, a HER-2 dimerization inhibitor, in patients with non-small cell lung cancer. Clin Cancer Res 12: 4436s–4440s 16857824 10.1158/1078-0432.CCR-06-0629

[bib10] Laux I, Jain A, Singh S, Agus DB (2006) Epidermal growth factor receptor dimerization status determines skin toxicity to HER-kinase targeted therapies. Br J Cancer 94: 85–92 16306877 10.1038/sj.bjc.6602875 PMC2361091

[bib11] Ng CM, Lum BL, Gimenez V, Kelsey S, Allison D (2006) Rationale for fixed dosing of pertuzumab in cancer patients based on population pharmacokinetic analysis. Pharm Res 23: 1275–1284 16715358 10.1007/s11095-006-0205-x

[bib12] Pinkas-Kramarski R, Shelly M, Glathe S, Ratzkin BJ, Yarden Y (1996) Neu differentiation factor/neuregulin isoforms activate distinct receptor combinations. J Biol Chem 271: 19029–19032 8702572 10.1074/jbc.271.32.19029

[bib13] Reid A, Vidal L, Shaw H, De Bono J (2007) Dual inhibition of ErbB1 (EGFR/HER1) and ErbB2 (HER2/neu). Eur J Cancer 43: 481–489 17208435 10.1016/j.ejca.2006.11.007

[bib14] Rowinsky EK, Donehower RC (1991) The clinical pharmacology and use of antimicrotubule agents in cancer chemotherapeutics. Pharmac Ther 52: 35–84 10.1016/0163-7258(91)90086-2 1687171

[bib15] Rustin GJ, Bast Jr RC, Kelloff GJ, Barrett JC, Carter SK, Nisen PD, Sigman CC, Parkinson DR, Ruddon RW (2004) Use of CA-125 in clinical trial evaluation of new therapeutic drugs for ovarian cancer. Clin Cancer Res 10: 3919–3926 15173101 10.1158/1078-0432.CCR-03-0787

[bib16] Sundaresan S, Roberts PE, King KL, Sliwkowski MX, Mather JP (1998) Biological response to ErbB ligands in nontransformed cell lines correlates with a specific pattern of receptor expression. Endocrinology 139: 4756–4764 9832411 10.1210/endo.139.12.6378

[bib17] Therasse P, Arbuck SG, Eisenhauer EA, Wanders J, Kaplan RS, Rubinstein L, Verweij J, Van Glabbeke M, van Oosterom AT, Christian MC, Gwyther SG (2000) New guidelines to evaluate the response to treatment in solid tumors. European Organisation for Research and Treatment of Cancer, National Cancer Institute of the United States, National Cancer Institute of Canada. J Natl Cancer Inst 92: 205–216 10655437 10.1093/jnci/92.3.205

[bib18] Walshe JM, Denduluri N, Berman AW, Rosing DR, Swain SM (2006) A phase II trial with trastuzumab and pertuzumab in patients with HER2-overexpressed locally advanced and metastatic breast cancer. Clin Breast Cancer 6: 535–539 16595039 10.3816/CBC.2006.n.009

